# Supplementary material for: The impact of social and environmental extremes on cholera time varying reproduction number in Nigeria
Source: PLOS Glob Public Health. 2022 Dec 14;2(12):e0000869. doi: 10.1371/journal.pgph.0000869 (PMC10022205; doi:10.1371/journal.pgph.0000869)
Supplement: S1 Text — The analysis includes R calculations, variable importance and model fitting for the full dataset. (DOC) [file pgph.0000869.s001.doc]

**S1 Text: Sensitivity analysis using confirmed and suspected cholera cases.** The analysis includes R calculations, variable importance and model fitting for the full dataset.

The data for the confirmed and suspected cholera cases had R calculated for 16 states (compared to 6 in the original model), which met the >40 cases thresholds for inclusion. The incidence and R calculations for the included states are shown below:

**R values over monthly sliding windows (line) calculated from the daily incidence (bar) of cholera.** The data used were suspected and confirmed cholera cases for 2018 and 2019 of states which met the threshold equal to or more than 40 cases.

The new dataset consisted of 5,627 data-points for variable importance and model fitting (compared to 279 in the original model). The variable importance plot (shown below) was similar to the variable importance for the original model (Fig.3), with only minimal changes in covariate importance order. This suggested that only small changes would be found in terms of the best fit model to the new dataset.

The new model did not improve model fit in terms of predictive power (shown below) and the same best fit model was selected. Any changes in the performance metrics were negligible (0.001 difference) and there was a slight decrease in correlation (0.71 in the new model), potentially due to the larger dataset creating greater variation. The sensitivity analysis using all the data, proved that the original model was robust and that the smaller dataset did not bias the results.

**The variable importance for the twenty-two covariates considered for model inclusion.** A serial interval of 5 days (with 8 days SD) was used and the numbers represent the clusters. SPEI01, 12, 48 - Standardised Precipitation Index calculated on 1, 12 and 48 month scale. PDSI - Palmers Drought Severity Index. MPI - Multidimensional Poverty Index. OCV – Oral cholera vaccination.

**Incidence-based vs covariate-based R values for the best fit model fitted to the testing dataset.** The error bars show mean absolute error and the line is a linear trend line.
