# Supplementary material for: The impact of social and environmental extremes on cholera time varying reproduction number in Nigeria
Source: PLOS Glob Public Health. 2022 Dec 14;2(12):e0000869. doi: 10.1371/journal.pgph.0000869 (PMC10022205; doi:10.1371/journal.pgph.0000869)
Supplement: S2 Text — (DOC) [file pgph.0000869.s002.doc]

**S2 Text: Additional covariate selection using linear regression**

The same 21 covariates (conflict, drought IDPs, WASH, healthcare, population and poverty) analysed using variable importance were also run through an additional covariate selection process and stepwise analysis as developed by:

1. Garske, T. et al. Yellow fever in Africa: estimating the burden of disease and impact of mass vaccination from outbreak and serological data. *PLoS Med*. **11**, e1001638 (2014).
2. Gaythorpe, K. A. M. et al. The global burden of yellow fever. *Elife* **10**, e64670 (2021).

The selection process removes covariates that are not significantly associated with the outcome variable (Rt3, Rt5, Rt8) at p = <0.1 using linear regression. It then clusters the remaining covariates based on the correction between them at an absolute pairwise correlation of above 0.75.

Ten were removed, either because they were not significantly associated with the outcome variable (R) or because they were too highly correlated with other covariates (healthcare facilities, piped water, open defecation, population, IDPs, severe poverty, vulnerable to poverty, basic hygiene). Eleven covariates remained and were grouped into five clusters, the clusters and variable importance of each covariate are shown below

**The variable importance for the eleven remaining covariates after variable selection.** All three serial interval values tested are shown (Rt3 - 3 days, Rt5 - 5 days, Rt8 - 8 days) and the numbers represent the clusters. SPEI01, 12, 48 - Standardised Precipitation Index calculated on 1, 12 and 48 month scale. PDSI - Palmers Drought Severity Index. MPI - Multidimensional Poverty Index.
