# Supplementary figures and images for: The impact of social and environmental extremes on cholera time varying reproduction number in Nigeria
Source: PLOS Glob Public Health. 2022 Dec 14;2(12):e0000869. doi: 10.1371/journal.pgph.0000869 (PMC10022205; doi:10.1371/journal.pgph.0000869)

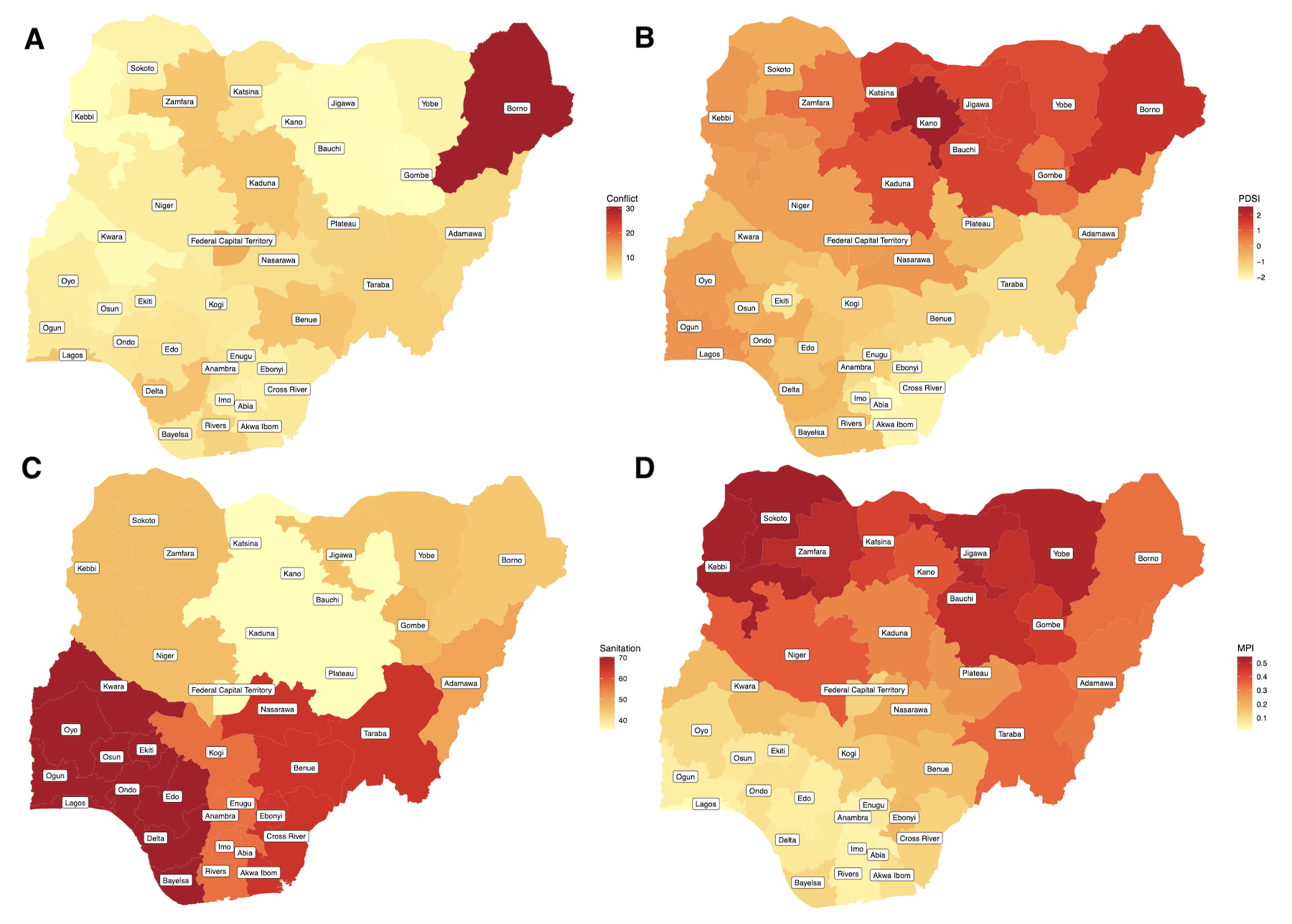

Supplement: S1 Fig — By state, covariates included: A, monthly conflict events, B, Palmers Drought Severity Index (PDSI), C, percentage access to sanitation and D, Multidimensional Poverty Index (MPI) [69]. (TIFF) [file pgph.0000869.s003.tiff]

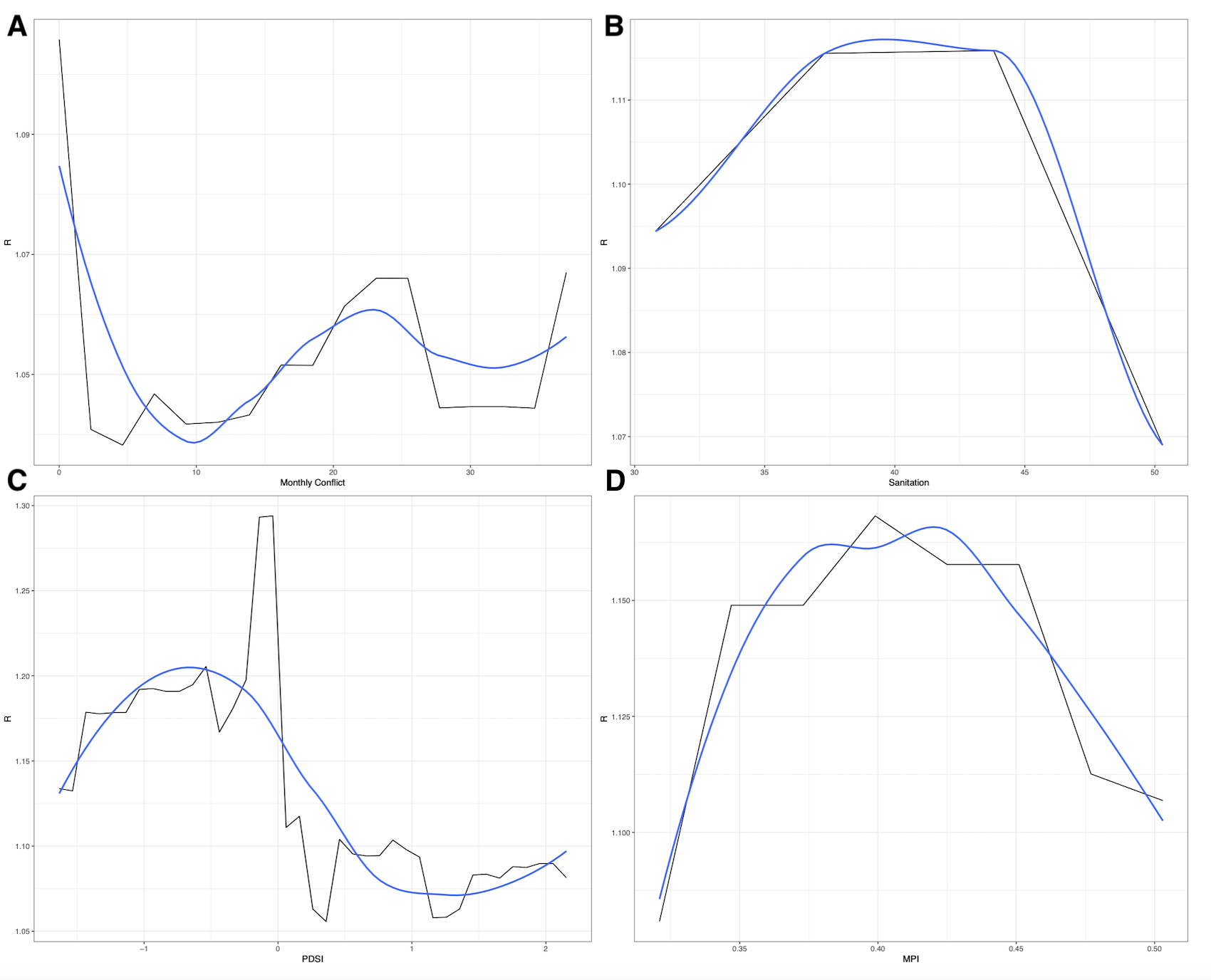

Supplement: S2 Fig — Showing the relationships between A, monthly conflict events, B, access to sanitation, C, Palmers Drought Severity Index (PDSI) and D, Multidimensional poverty Index (MPI) and R. (TIFF) [file pgph.0000869.s004.tiff]

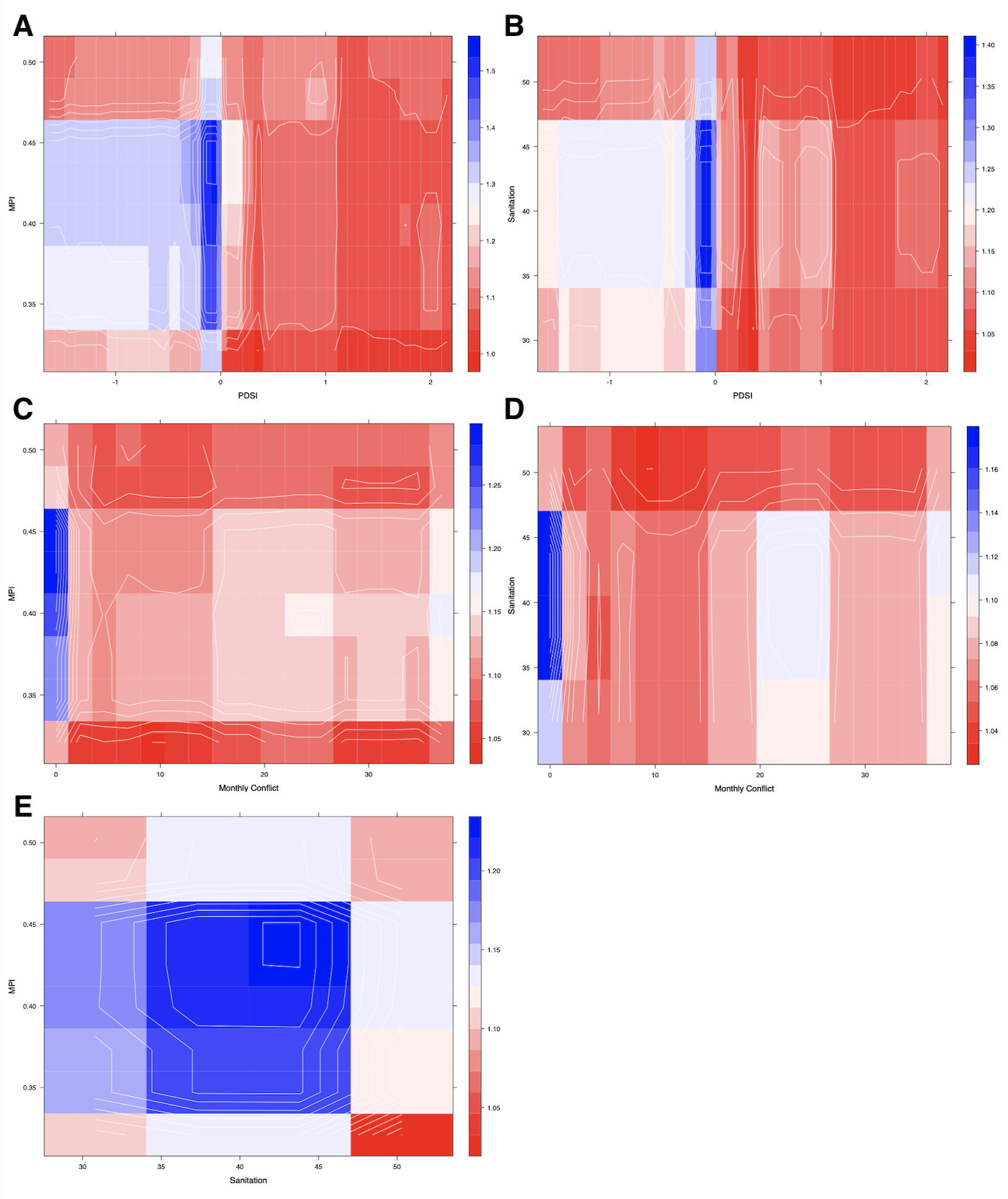

Supplement: S3 Fig — Showing the relationships between A, Palmers Drought Severity Index (PDSI) & Multidimensional poverty Index (MPI), B, PDSI & Sanitation, C, Monthly conflict & MPI, D, Monthly conflict & Sanitation, E, Sanitation & MPI and R. (TIFF) [file pgph.0000869.s005.tiff]

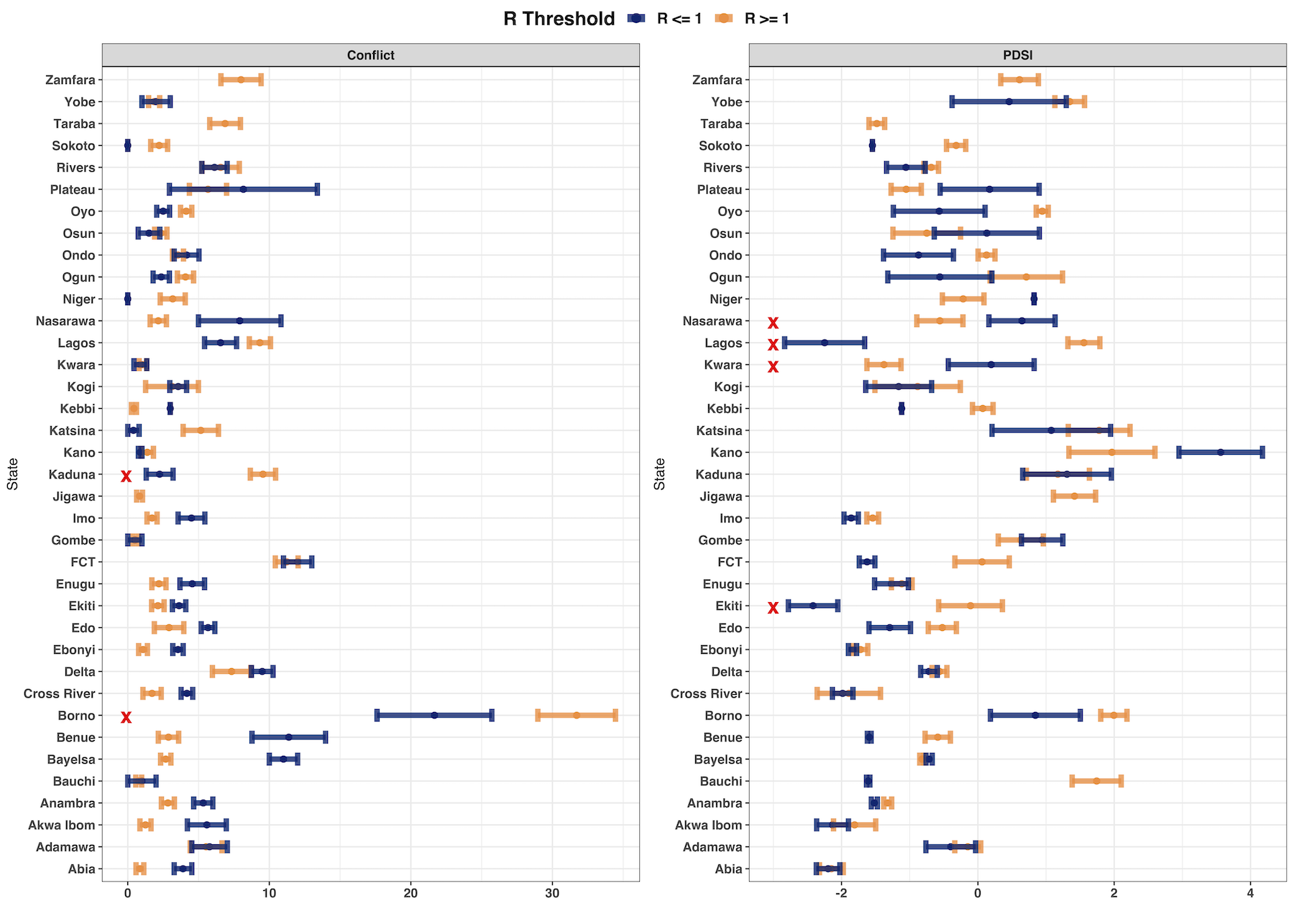

Supplement: S4 Fig — The mean and standard error for the two covariates for the full dataset split by state and R threshold. The red “x” shows the states which were included in the sub-national analysis: Conflict (Borno and Kaduna), extreme wetness (Lagos and Ekiti), extreme dryness (Nasarawa and Kwara). (TIFF) [file pgph.0000869.s006.tiff]

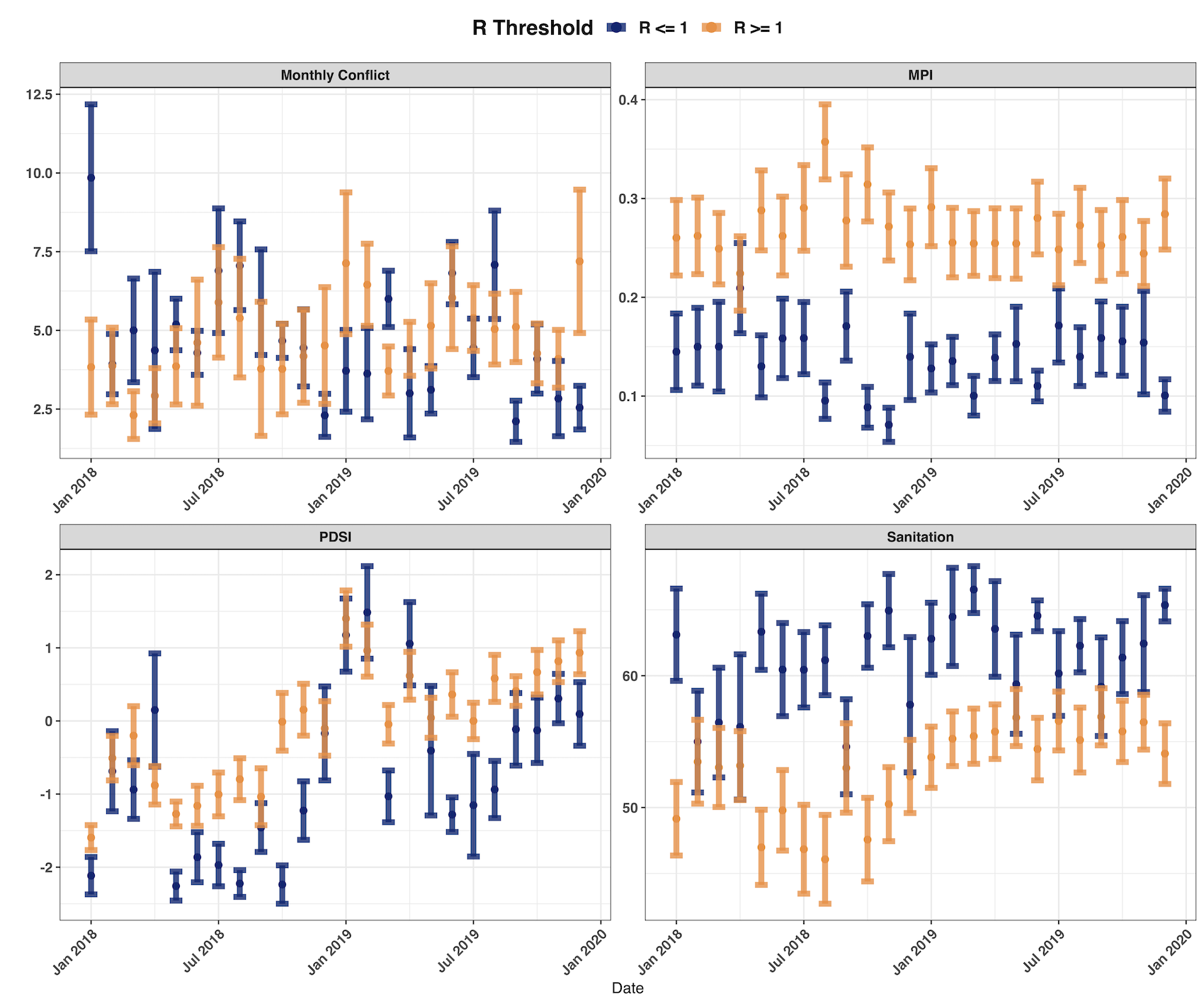

Supplement: S5 Fig — The mean and standard error for the four covariates included in the best fit model for the full dataset split by month and R threshold. (TIFF) [file pgph.0000869.s007.tiff]

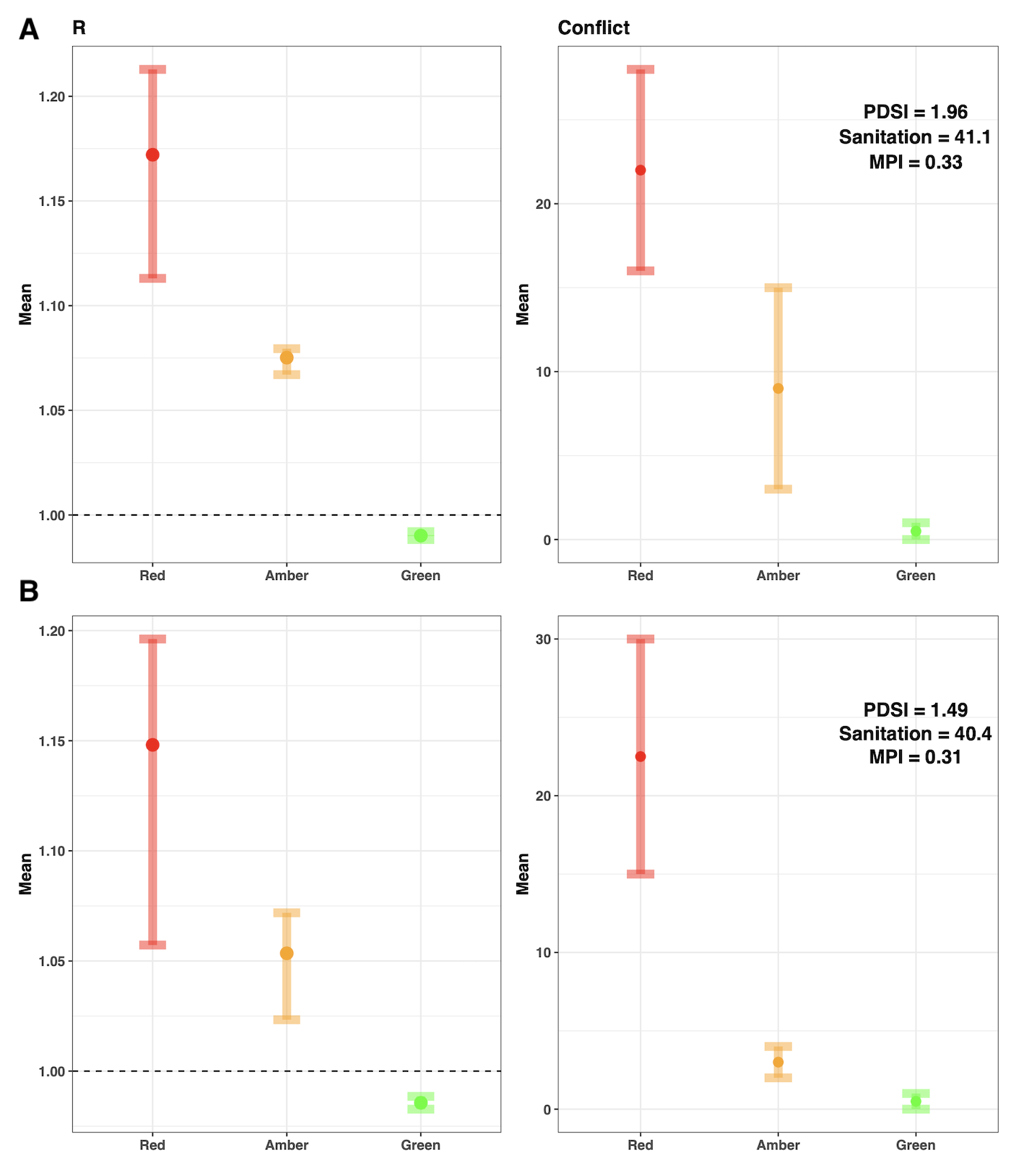

Supplement: S6 Fig — The other three (PDSI, Sanitation and MPI) covariate values were retained at the mean value for R = >1 for the full dataset (values shown in the plot) for A, Borno and B, Kaduna. (TIFF) [file pgph.0000869.s008.tiff]

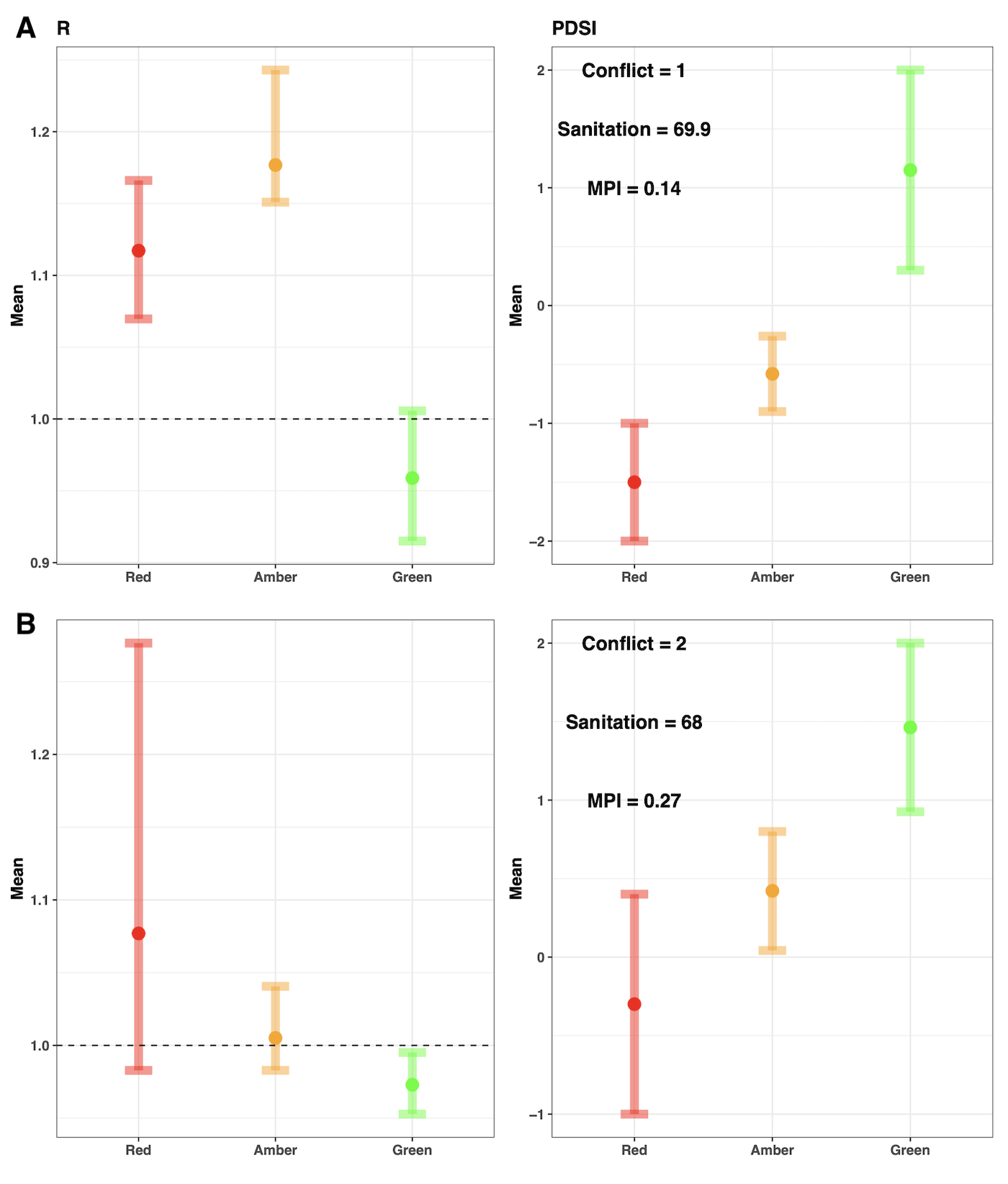

Supplement: S7 Fig — The other three (Conflict, Sanitation and MPI) covariate values were retained at the mean value for R = >1 for the full dataset (values shown in the plot) for A, Kwara and B, Nasarawa. (TIFF) [file pgph.0000869.s009.tiff]

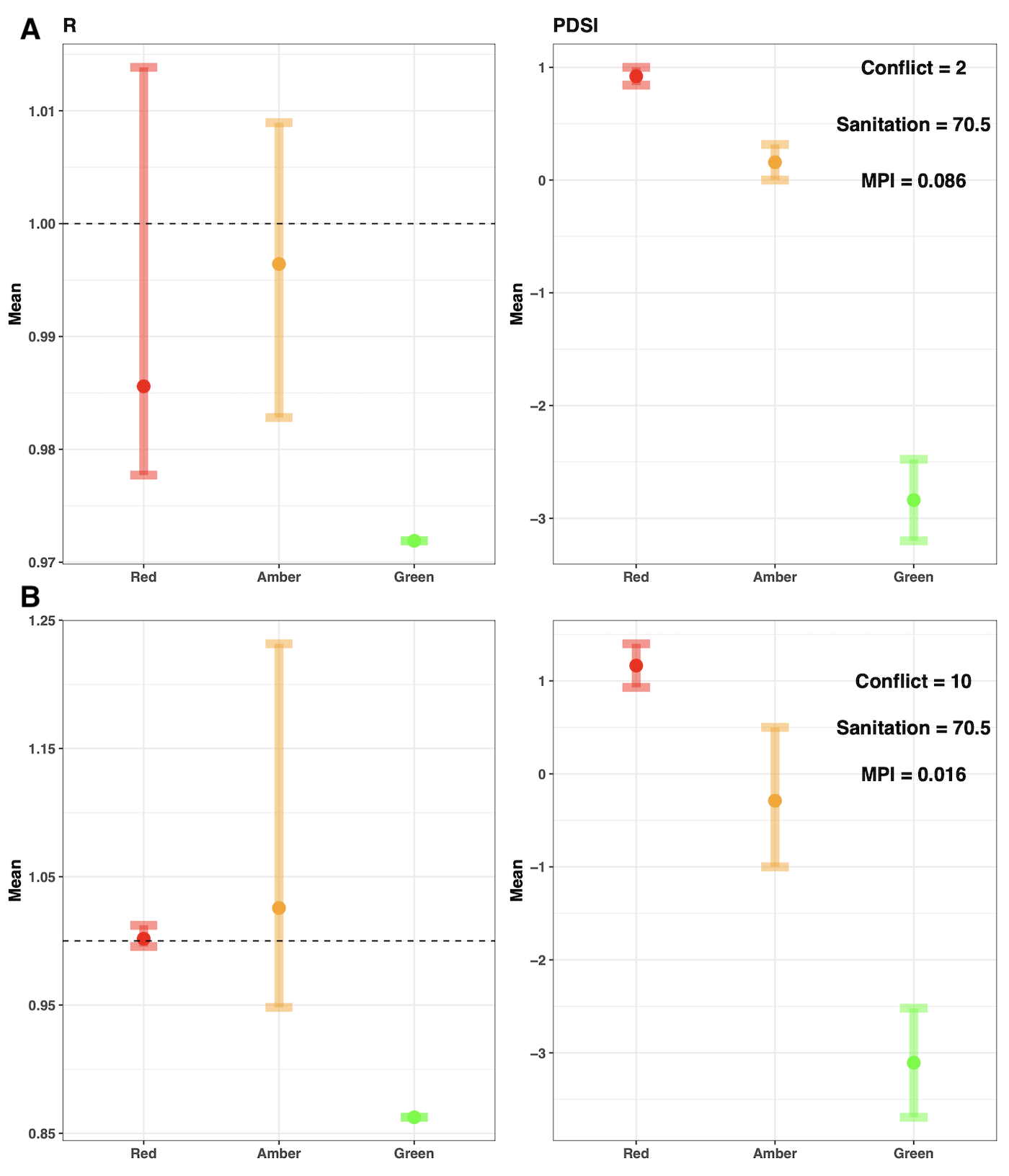

Supplement: S8 Fig — The other three (Conflict, Sanitation and MPI) covariate values were retained at the mean value for R = >1 for the full dataset (values shown in the plot) for A, Ekiti and B, Lagos. (TIFF) [file pgph.0000869.s010.tiff]
