# Supplementary material for: The impact of social and environmental extremes on cholera time varying reproduction number in Nigeria
Source: PLOS Glob Public Health. 2022 Dec 14;2(12):e0000869. doi: 10.1371/journal.pgph.0000869 (PMC10022205; doi:10.1371/journal.pgph.0000869)
Supplement: Attachment — Submitted filename: Review Response.docx [file pgph.0000869.s011.docx]

**Response to Reviewers - The impact of social and environmental extremes on cholera time varying reproduction number in Nigeria**

We would first like to thank the Editor and the reviewers for their time in reviewing our manuscript and providing many constructive comments and suggestions, which we feel has helped improve our contribution.

We welcome the interest and positive responses to the work presented in terms of manuscript structure and presentation, along with the methodology chosen here.

In our response and revisions, we have taken note of all the feedback and in particular have:

- Improved our communication of the novelty of the findings
- Elaborated further upon the strengths and limitations of the datasets
- Strengthened the discussion to incorporate or expand some important points noted by the reviewers

Point-by-point responses are provided below. Responses to reviewer comments are in *italics*. For the correct line numbers tracked changes need to be changed to “no” or “simple” markup.

**Editor**

Amend online detailed Financial Disclosure statement - Please state the initials, alongside each funding source, of each author to receive each grant.

*“This work was supported by the Natural Environmental Research Council [NE/S007415/1] (https://www.ukri.org/councils/nerc/), and the grant was awarded to G.Charnley as part of the Science and Solutions for a Changing Planet DTP. G.Charnley also acknowledges funding from the MRC Centre for Global Infectious Disease Analysis [MR/R015600/1], jointly funded by the UK Medical Research Council (MRC) and the UK Foreign, Commonwealth & Development Office (FCDO), under the MRC/FCDO Concordat agreement and is also part of the EDCTP2 programme supported by the European Union. The funders had no role in study design, data collection and analysis, decision to publish, or preparation of the manuscript.”*

*The above sentence was re-submitted*

Please provide separate figure files in .tif or .eps format only and ensure that all files are under our size limit of 10MB.

*All figures have been re-submitted after conversion to a .tif file format, and meet the size limits of the journal*

Add a full list of legends for your Supporting Information files after the references list.

*A list its now included at the end of the references, as requested*

Figure 1a, Figure 5, and S1 Figure: please (a) provide a direct link to the base layer of the map (i.e., the country or region border shape) and ensure this is also included in the figure legend; and (b) provide a link to the terms of use / license information for the base layer image or shapefile. We cannot publish proprietary or copyrighted maps (e.g. Google Maps, Mapquest) and the terms of use for your map base layer must be compatible with our CC-BY 4.0 license.

*The file used for all three maps are from the same source, The World Bank Data Catalog. (*[*https://datacatalog.worldbank.org/search/dataset/0039368*](https://datacatalog.worldbank.org/search/dataset/0039368)*). The license is a CC-BY 4.0, allowing the shape file to be used, modified and distributed, given appropriate credit (*[*https://datacatalog.worldbank.org/public-licenses?fragment=cc*](https://datacatalog.worldbank.org/public-licenses?fragment=cc)*). The source of the file has been referenced in all three figure legends.*

Certain points should be better presented:

Role of environmental factors and the mechanisms by which they act.

*These mechanisms and risk factors are in the Introduction at lines and the lines 61-66 Discussion at lines 434-435.*

The influence of the relatively short length of the data used should also be better discussed

*The data used here are detailed surveillance data which are very time consuming to collect and collate into larger datasets. Both the strengths and limitations of the data, and why this data source is so important for the modelling completed here, has been discussed further in the Discussion at lines 456-458 and 466-477. The influence of the relatively short time length of data may have missed certain effects/relationships and means that caution is needed when making generalizable conclusions about historical cholera in Nigeria.*

Place the results presented in the context of current knowledge about cholera epidemics by	emphasizing more clearly the novelty of the results presented.

*Novelty of the results, specificity of the results, the use of reproductive number (R) and machine learning methods and previous research have been elaborated on throughout the Introduction and Discussion sections in line with the Editor and reviewer comments (lines 68-71, 90-100, 380-383, 401-403, 417-421, 486-489).*

**Reviewer #1**
The conclusions drawn by the authors are already well known and described in the literature about the reducing effect of increase sanitation and decrease poverty on the cholera transmission as well as the effect of extreme climatic events as floods or droughts, or conflicts.

*We agree that some of these cholera risk factors are already well established. However, cholera is one of the oldest known diseases and remains one of the biggest contributors to under 5 and diarrhoeal disease mortality. Mortality is particularly high in fragile settings, suggesting that further research, quantification and emphasis on disaster-related cholera outbreaks are needed. We contend that the novelty of our results, which distinguish them from previous studies, is our chosen methodology, accurate data sources, and specific social and environmental risk factor thresholds in the context of Boko Haram and environmental change in Nigeria.*

Furthermore, this study does not consider prevention interventions or vaccination campaigns.

*Despite not being referred to specifically as an intervention, our deep consideration of factors related to sanitation and access to care clearly connects to the most common and impactful intervention actions relied upon during cholera outbreaks. Healthcare was nevertheless not selected in the best fit model, indicating that other factors are more explanatory in terms of exacerbating risk of cholera outbreaks. Other data described as specific interventions were not available but the information on sanitation (as healthcare was not selected in the model) as a relevant intervention was discussed at lines 402-404.*

*Some vaccination coverage data were available through NCDC, but originally, we did not fit the data to the model due to the data limitations and therefore assumptions that would be needed (e.g., target population). The data were represented as state, start date of campaign, end date and vaccination coverage. We transformed coverage to a binary outcome variable for a specific year (e.g., if there was 100% vaccination coverage in that year, the state was assigned a 1) and used this in the covariate selection process. Details of the analysis are available in the Methods at lines 109-112. In summary, OCV formed a distinct cluster and therefore could be considered in isolation for model inclusion, it also had relatively high variable importance (see updated Figure 3), but when included in the best fit model, the improvements in fit were negligible (0.001 improvements in RMSE and R²), therefore it was not included due to the limitations and assumptions in assigning it as a binary outcome.*

But the study limitations negatively impact the value of the results (2 years dataset, low number of cholera cases).

*We agree the duration of the dataset is not without some limitations in terms of our ability to draw more generalisable conclusions about cholera globally and historically in Nigeria but contend that is does not in itself discount the value of the results given the appropriate analytical methods and degree of caution we brought in the interpretation of results (as is the case for all time-series data).*

*We also contend that this dataset has instead several key advantages that further justify its use with our chosen approach.*

*In terms of low case numbers, we further note that Nigeria is one of the most affected countries globally and as such these data are uncharacteristically rich compared to many other settings. Unfortunately, cholera cases are often not confirmed, especially during outbreaks or in fragile settings. Unconfirmed cases were therefore removed, as Nigeria also has a high burden of several other diarrhoeal pathogens, which are likely to be reported as cholera and which therefore represented potential confounders that we aimed to neutralise.*

*We have now further elaborated on these issues and advantages in the Discussion at lines 456-460 and 466-477.*

*Specifically, due to the epidemiological features of cholera (short incubation, rapid onset, immediate mediating effects of various risk factors) we placed emphasis on data accuracy over data volume. We used the best possible data currently available, which were surveillance data that are both time consuming to collect and presents many challenges in fragile settings, but which are nevertheless relatively limited in terms of temporal coverage. Instead of seeing this as a limiting factor, we instead applied an appropriate and novel method of analysis in turning to machine learning models, well known for their lack of assumptions (compared to e.g., linear modelling) and ability to maintain predictive power given so called small N (sample size) and large K (predictor variables). Larger publicly available data sources are commonly used in cholera modelling, but we argue that our approach fills a critical gap and expands further the datasets and tools available to researchers to try to understand and manage cholera.*

I don’t agree with the authors hope this study shows the importance of doing so to gain a more accurate understanding of disease outbreaks in complex emergencies. What this study could help is to draw a picture at a given time for in the specific context of cholera in Nigeria at that time by a quantitative approach. It can serve to highlight specific vulnerabilities to elaborate specific recommendations to the public health authorities and political decision makers for the disease prevention.

*We thank the reviewer for this recommendation in terms of how the work presented here could be useful and impactful and have addressed this in the response above and in the Discussion at lines 456-460 and 466-477.*

*Additionally, the very specific recommendations and conclusion that can be made through these results, which are a strength of this study, have now been elaborated on at lines 381-383 and 398-400.*

But in the context of an emergencies, when some health authorities already struggle with the case detection, they put human and financial efforts on outbreak responses in the field instead of complex modeling.

*We agree and do not imply that there is competition between funding responses post disaster vs modelling, or that any diversion of limited funds should take place. Health authorities, as noted, are often stretched during outbreaks and other emergencies. Here, our focus is on developing and compiling datasets and tools that could aid in preparedness as well as response in terms of outbreak prevention and amelioration, which could include service prioritisation (e.g., WASH). As discussed, our results are specific to a certain time and place, but one of the main and higher-level conclusions of this research is how reducing poverty and sanitation can reduce the impact of these disasters, allowing people to adapt and reduce outbreak risk.*

**Reviewer #2**

Although the study attempts to related both environmental and socio-economic extremes to cholera outbreaks, in reality, the paper mostly focuses on the social, political, and economic forcing and barely on environmental extremes.

*Thank you for pointing out this potential issue in the balance of our manuscript in terms of environmental vs social discussion points. Here, three social/political factors were selected in the best fit model, vs only one environmental, which is why the discussion is more heavily focused on these factors. Specific paragraphs in both the Results and Discussion are designated to discussing PDSI in detail (lines 344-352 & 426-441). The results found (as in other modelling studies) that social factors were more important in the model and decreasing pre-existing social, political and economic vulnerably, reduced the impact of environmental extremes.*

The environmental variables are not properly developed. PDSI is a variable that is most appropriate to reflect long term change – and not really a daily process - only two years of daily data seems insufficient for understanding the trajectory of extreme droughts with daily scale cholera risk / reproductive ratio.

*Thank you for your suggestion in terms of environmental variable development, which we agree could have been expanded on more in the manuscript. A full explanation for the use of PDSI and SPEI are now delineated in the Methods at lines 123-129 and Discussion at lines 435-441. Despite some data being measured on a daily temporal scale (e.g., daily conflict events), no data in the best fit model were on a daily temporal scale and R was calculated over a monthly sliding window, not daily. The PDSI and SPEI data were both measured monthly and a more detailed description of the temporal granularity of the data used for each variable are now included in the Methods section (lines 114-121 and 131-135).*

In addition, the mechanisms or pathways of how the environmental variables might play a role in cholera transmission or reproductive ratios – is virtually missing. I assume there is an underlying association with water scarcity and cholera that is implied here. But the reproductive number is a variable that can be affected in many facets of water scarcity – none of which have been envisioned, explained or discussed in this manuscript.

*Mechanisms for water scarcity resulting in cholera outbreaks were suggested in the Discussion at lines 434-435 and some additional information has been added to the Introduction at lines 64-66. As suggested, some environmental mechanisms here are assumed, including the multifaceted nature of them, and have been the sole focus of previous research which is referenced in the manuscript (ref [7]).*

The reproductive number itself is a simulated variable here, although simulated in two different ways. But there is no way to validate the patterns without looking at pathways. Especially, the simulations over the western states with such little data, except Borno and Adamawa, are very hard to justify.

*As addressed above (in response to the Editor and review 1), it was more important when using machine learning methods such as random forest to fit the model to accurate data, than to use a longer time-series with more cases but with less accurate data points. More states could have been included in the R calculation from the incidence data, but this created a high level of uncertainty and meant that the coefficient of variation threshold was not met (line 161). By instead fitting a smaller but more accurate dataset, we believe we have been able to generate more robust model predictions. This is presented through the correlation between incidence and covariate-based R values and through the prediction of high R values in states that have known high cholera burdens or areas which only just missed out on the 40 case thresholds (discussed at lines 301-304). Limitations of the dataset both in terms of cases, timescale and spatial extent have been discussed further in the Discussion at lines 435-441, 456-458, and 466-477.*

The above-mentioned issue with calculating reproductive ratios are reflected through the limited correlation seen between covariate and incidence-based R calculations.

*The correlation between the covariate and incidence-based R calculations is 0.87, for a correlation coefficient this is very high (perfect positive correction being 1). It is particularly encouraging considering the model was fit to a limited dataset and further shows the strength of the data used here. The line of best fit in Figure 4 illustrates this strong linear relationship very well.*

The threshold values around R = 1 are also not clear. There should be clear guidelines what those thresholds are for the dry or the saline conditions.

*The reasons for why specific guidelines were not used around R thresholds are explained in the Methods at lines 234-237. Specifically, the complexity of the relationships between the four covariates and R here meant that a pre-defined threshold was not realistic and could have resulted in the extent of the relationships being missed. Therefore, the full range of values for each covariate in the scenario were included in the Results (expanded at lines 229-232). As previously pointed out in the feedback, making generalisable conclusions about dry/wet conditions for each state, across the country, is problematic given the limitations discussed and making a specific guideline would exacerbate this issue.*
