# Supplementary material for: The impact of social and environmental extremes on cholera time varying reproduction number in Nigeria
Source: PLOS Glob Public Health. 2022 Dec 14;2(12):e0000869. doi: 10.1371/journal.pgph.0000869 (PMC10022205; doi:10.1371/journal.pgph.0000869)
Supplement: Attachment — Submitted filename: Review Response2.docx [file pgph.0000869.s012.docx]

**Response to Reviewers 2 - The impact of social and environmental extremes on cholera time varying reproduction number in Nigeria**

Thank you to the Editor and the reviewers for their time in reviewing a second revision of our manuscript and providing additional comments and feedback, which we believe has further strengthened our manuscript.

In our response and revisions, we have focused on further addressing the comments raised regarding the timescale/quantity of the data and the interpretation of the results.

Point-by-point responses are provided below. Responses to the Editor and reviewers are in *italics*. For the correct line numbers, tracked changes need to be changed to “no” or “simple” markup.

**Editor**

*As requested, the research ethics statement was moved to the beginning of the Methods sections and given a sub-heading. Details were also given as to why formal consent was not obtained.*

*“The datasets and methods used here were approved by Imperial College Research Ethics Committee and a data sharing agreement between NCDC and the authors. Formal consent was not obtained for individuals in the data used here, as the data were anonymised.”*

**Reviewer #1**

*Thank you for your feedback on our manuscript and for your time in reviewing the revisions.*

**Reviewer #2**

*Thank you for your feedback to help further strengthen our manuscript regarding the methodology and interpretation here.*

The authors have not advanced their case with respect to the variable selections, the model results, and the claimed conclusions. Machine Learning (ML) ‘can’ be a powerful tool – if it has enough good data to train the model – which is not the case here. Just using Machine learning cannot be the end itself. Thus, while focusing on a smaller high quality confirmed dataset has its merits – it also lends to the conclusion that ML is a good choice here.

The ML will fit to whatever you give it to fit. If you fit a smaller dataset, it will bias itself to that specific scenario. Using that ‘best fit’ model to predict the R values of other reasons seems a very erroneous approach to begin with. And there are little data to validate these other regions. High correlation can also result from a smaller number of data points, we all understand that.

*We believe that the original model, fit to a dataset of 279 data-points at 70% training, was sufficient to avoid bias. If the dataset was too small, this would have resulted in over-fitting and little to no error in the predictions, which was not the case here (lines 219-223 and Fig.4). To provide further evidence for this decision to use only confirmed cases and how using the most accurate (albeit smaller) dataset improved our model robustness. We re-ran our models using all the data, including suspected and confirmed cases.*

*The new model was fit to 5,627 data-points, across 16 states (vs 6 in the original model). We re-ran the variable importance analysis, creating a new Figure 3 for comparison. The variable importance was similar to the original model, with some covariates slightly more important but generally following the same order. To account for these changes, we re-fit the model, stepping through model possibilities and adding additional covariates that were more important in the new variable importance plot (e.g., healthcare metrics).*

*The new model did not improve model fit in terms of predictive power and the same best fit model was selected. Any changes in the performance metrics were negligible (0.001 difference). The new analysis using all the data, proved that our original model was robust and that the smaller dataset did not bias the results. Therefore, we kept our original model, as we believe the quality of the data is very important here, particularly given the issues with cholera data that are known and discussed in the manuscript. We included the additional analysis with all the data in the Supplement (S1 Information) and stated this in the manuscript at line 114 and 262.*

*In the previous revision, we agreed that the quantity and timescale of our dataset was a limitation and incorporated further limitations and explanations into our manuscript.*

Even if we are confident Random Forest is the way to go … the ranking of the variables in both the primary and the secondary selection process lead to the environmental variables (PDSI and other SPEI indices). The MPI, socio-economic, or sanitation related variables are all way down the list.

*Only one covariate from each cluster can be fit to the model to account for multi-collinearity, and Figure 3 is not used as a list for model fitting. The model with the best predictive accuracy, included the covariates which were the highest in terms of variable importance for each cluster. All covariates were tested and those with the lowest variable importance did not improve model fit, as expected. Although the drought indices are at the top, this is not considering the dependencies/relationships between the covariates within the best fit multi-variate model.*

While the model has its findings, the authors have not interpreted or conveyed it correctly. Sanitation coverage is universally low in Nigeria. How the role of the droughts make the pathogen transmit is the real story here!

*Sanitation is not universally low in Nigeria. In most southern states access to improved sanitation is around 70% coverage and up to 90% have an improved drinking water source (see DHS datasets and S1 Figure). To dismiss Nigeria as having a universally low access to WASH, is dismissing these development gains and would not be highlighting the findings as the data suggests.*

*The regional inequities in Nigeria are where the conclusions from the results here are made. Drought is a global issue, affecting countries of high-, middle- and low-income. Whereas cholera is not, only causing large outbreaks in countries without sufficient access to sanitation, hygiene and safe drinking water. Despite the role of drought being important here, we do not believe that drought is the main driver, and our results confirm this, as described in the Discussion:*

*“For Borno, raising monthly conflict events from 1 to 2 increased R above 1, but an increase in access to sanitation from 41-46% pushed the R value back below one. This relationship continued in a stepwise pattern and in a similar way for MPI and drought but to a lesser degree.”*

*“Four states were investigated to evaluate the differences between extreme wetness and extreme dryness and R values over 1. In contrast to Borno and Kaduna, all four states predicted consistently low R values, a potential explanation for this is the high variable importance of PDSI (Figure 3) and the high levels of sanitation and low levels of poverty in all four states, contributing to overall lower predicted levels of cholera.”*

*If drought was the main mechanism driving cholera in Nigeria, then these states would have predicted much higher R values, regardless of sanitation and poverty, but this was not the case. Furthermore, as stated in the manuscript we found both extreme wetness and extreme dryness to impact the R values, and therefore to discuss drought alone as the driver of cholera would not be an accurate representation of our results. Additionally, if these methods were run in a country which did have universally low access to sanitation, this is not to say that sanitation would not be important in the model.*

Please highlight the main findings of the models as the data suggest and not the way they help fit a narrative. Similarly, the added sentence in the discussion “The results here suggest that the expansion of sanitation would be particularly impactful or cholera control for those in states with <50% access, which currently includes all northern states.” Is almost universal. Please bring forward the discussion on the droughts (and water scarcity) play a central role in cholera progression in Nigeria … where safe water and sanitation is universally low to begin with.

*Please see the comments above in terms of sanitation coverage in Nigeria and the role of	drought. Further explanation to the interpretation of drought is below:*

*We agree that drought and the mechanisms for which drought and water scarcity can lead to cholera outbreaks are important. Drought, water scarcity and cholera have been the focus of previous research on longer datasets, see references 7 and 11 and briefly mentioned in the Discussion: “Mechanisms through which this can occur includes a lack of water increasing risky drinking water behaviour and floods allowing for the dispersal of the pathogen [7,65].”*

*In the previous revision we addressed the comment:*

*“The environmental variables are not properly developed. PDSI is a variable that is most appropriate to reflect long term change – and not really a daily process - only two years of daily data seems insufficient for understanding the trajectory of extreme droughts with daily scale cholera risk / reproductive ratio.”*

*Based on this comment, we included this as a limitation and drought has not been the main focus here. As correctly pointed out, drought is a long-term process and due to the limited timescale of the data, conclusions regarding drought may be over-estimated or over-simplified from these results (lines 439-445). To draw conclusion on drought and water scarcity from our results would be presenting our results to fit a narrative we cannot determine from two years of monthly data.*

*Improving local water and sanitation access will help only if Nigeria can handle the extreme events such as droughts*

*We agree that how the population responds to an extreme event is very important for cholera and other diseases. Nigeria’s ability to handle the extreme event will depend upon their ability to adapt. This is determined based upon pre-existing vulnerabilities, such as water and sanitation access. Reducing these vulnerabilities will reduce the risk of pathogen exposure, regardless of the environment.*
